# Preventing Biofilm Formation by Dairy-Associated Bacteria Using Peptide-Coated Surfaces

**DOI:** 10.3389/fmicb.2019.01405

**Published:** 2019-06-26

**Authors:** Alon Friedlander, Sivan Nir, Meital Reches, Moshe Shemesh

**Affiliations:** ^1^ Department of Food Sciences, Institute for Postharvest Technology and Food Sciences, Agricultural Research Organization, The Volcani Center, Rishon LeZion, Israel; ^2^ Institute of Dental Sciences, The Hebrew University-Hadassah, Jerusalem, Israel; ^3^ The Institute of Chemistry and the Center for Nanoscience and Nanotechnology, The Hebrew University of Jerusalem, Jerusalem, Israel

**Keywords:** antifouling peptide, biofilm formation, dairy industry, bacterial contamination, milk, dairy food

## Abstract

Biofilm-forming bacteria, which colonize the surfaces of equipment in the dairy industry, may adversely affect the safety and quality of the milk and its products. Despite numerous efforts to combat biofilm formation, there is still no effective technological means to thoroughly solve the biofilm problem in the dairy industry. Here, we introduced peptide-based coating in order to modify the physical properties of the stainless steel surface by affecting its availability for bacterial adhesion. We found that the coated surface displays a notable decrease in the ability of bacterial cells to attach and to subsequently form biofilm by Gram-positive *Bacillus licheniformis* and Gram-negative *Pseudomonas aeruginosa.* Furthermore, the coated surface retained its anti-biofilm ability following its exposure to raw milk. Importantly, the modified surface did not affect the milk coagulation process or its nutritious properties and quality. Overall, this anti-biofilm approach may serve as an attractive solution for the dairy industry in its struggle against bacterial contamination.

## Introduction

Biofilm-forming bacteria are known to be a major source of both spoilage and pathogenic microflora in the dairy industry (Elmoslemany et al., 2009). Therefore, bacteria that form biofilms may adversely affect the safety and quality of milk and its products (Moretro and Langsrud, 2017). The main source of contamination of dairy products is often associated with the formation of biofilms on the surfaces of milk transport pipes, milking containers, and accessories in the dairy industries (Srey et al., 2013). Biofilms are established mainly under conditions that allow bacteria to easily adhere to the walls of the pipes, for example, when the milk is found in transport pipes without flow (Stoodley et al., 2002). The bacteria may detach from biofilms and contaminate the milk as it passes surfaces (Austin and Bergeron, 1995). Moreover, biofilm bacteria can also increase the corrosion of metal pipes, reduce heat transfer, and increase fluid frictional resistance (Kumar and Anand, 1998; Gupta and Anand, 2018).

Many attempts have been made to combat biofilm formation in the dairy industry, including cleaning and thoroughly disinfecting surfaces that are exposed to milk during its processing (Bremer et al., 2006). However, antimicrobial treatment may be compromised since the disinfectants do not penetrate the biofilm’s matrix that is mounted on the surface (Simoes et al., 2006).

One of the possible strategies to combat biofilm formation is by preventing bacterial adhesion to surfaces in advance (Palmer et al., 2007). This strategy includes either reducing surface roughness, antimicrobial coatings, or anti-adhesive compounds that repel bacterial cells by means of physical mechanisms (Verran and Whitehead, 2005; Lejars et al., 2012). Owing to the need for nontoxic anti-biofilm materials, coating surfaces with anti-adhesive agents is more attractive because it does not involve toxic compounds and is not likely to cause resistance development in the bacterial flora (Nir and Reches, 2016).

Biofilm formation initiates with the attachment of bacterial cells to the surface; it involves the synthesis of a protective extracellular matrix, which allows the bacteria to survive under hostile environments (Hall-Stoodley et al., 2004; Chen et al., 2007). The initial adhesion process depends on bacterial species, the interaction medium, and surface properties (Pereni et al., 2006). The surface properties can be designed and modified to reduce bacterial adhesion. Surface modification refers to the alteration of the physical and chemical properties of the substrate (roughness, hydrophobicity, and more) that lead to an intervention in bacterial attachment and biofilm formation (Kasimanickam et al., 2013).

One of the promising ways of modifying surfaces involves the use of antimicrobial peptides. These peptides are a heterogeneous group of small molecules produced by a wide variety of cells that exhibit potent antibacterial activity (Batoni et al., 2011). Some peptides are characterized by their ability to inhibit the biofilm’s formation (Sakala and Reches, 2018); therefore, many attempts have been made to use them in their natural or synthetic form in both the medicine and food industries (Geng et al., 2018). These peptides influence bacterial cellular processes and, therefore, bacteria may develop resistance, which occurs in the case of antibiotics (Chung and Khanum, 2017; Hamley, 2017).

Previous studies showed that peptides could modify surfaces to attain anti-biofilm properties (da Silva et al., 2017). Maity et al. (2014) showed that the antifouling properties of surfaces modified with a tripeptide could inhibit biofilm formation. This peptide, DOPA-Phe(4F)-Phe(4F)-OMe, consists of 3,4-dihydroxy-L-phenylalanine (L-DOPA), which can adhere to various surfaces (Sedo et al., 2013) and two amino acids of phenylalanine with fluorinated residues, which direct their self-assembly onto a surface and alter the surface properties, thus preventing the attachment of the proteins and bacteria (Reches and Gazit, 2003; Maity et al., 2014; Alves et al., 2018). In addition, it was recently demonstrated that the peptide coating is not toxic to mammalian cells (Yuran et al., 2018).

Here the use of this tripeptide-based coating is reported under conditions relevant to the dairy industry. We show how the peptide coating affects the attachment and growth of two different bacterial species, *Bacillus licheniformis* (a Gram-positive bacterium) and *Pseudomonas aeruginosa* (a Gram-negative bacterium), which are considered extremely problematic species in this industry. Moreover, we studied how this coating affects the technological properties (e.g., the protein level, clotting parameters) of dairy products.

## Materials and Methods

### Materials

Stainless steel 316 l was obtained from Holland-Moran LTD (Yehud, Israel). The tripeptide was synthesized by solution-phase synthesis, as reported in previous work (Maity et al., 2014). Three percent fat homogenized ultra-high temperature processing (UHT) milk was obtained from Tnuva (Rehovot, Israel). Raw milk was obtained from the dairy farm of the Agricultural Research Organization (ARO), (Rishon LeZion, Israel). Skimmed milk was obtained from Difco (Sparks, USA). Rennet enzyme was purchased from Gist-Brocades (Delft, The Netherlands).

### Strains and Growth Media

Two bacterial species were used for this study: Gram-positive *Bacillus licheniformis* strain S127, which was isolated from a sheep udder clinical infection (Ostrov et al., 2019) as well as a Gram-negative *Pseudomonas aeruginosa* PA14 strain (Pechook et al., 2015). For routine growth, both strains were propagated in a Lysogeny broth (LB; Bacto, Le Pont de Claix, France) or on a solid LB medium supplemented with 1.5% agar. For analysis of submerged biofilm formation, *B. licheniformis* was grown on either peptide-coated or uncoated stainless steel surfaces (used as a control) in tryptic soy broth (TSB; Bacto, Le Pont de Claix, France) with 0.5% yeast extract, whereas *P. aeruginosa* was grown in LB medium.

### Preparation of Peptide-Coated Surfaces

Prior to coating, the surfaces (1 cm × 1 cm) were sterilized by dipping them in pure ethanol. Later, the surfaces were placed into 3-ml glass vials and dipped in peptide solution (0.5 mg/ml in ethanol, 700 μl) overnight. After incubation, the surfaces were washed with ethanol and dried under nitrogen gas. For a reference (uncoated surface), the tested substrates were dipped only in ethanol, to be used as a control.

### Water Contact Angle

A Theta Lite optical tensiometer (Attension, Finland) was used to measure the water contact angle of the samples, ensuring that the coating process successfully modified the surface. Measurements were conducted in triplicates and the values were averaged.

### X-Ray Photoelectron Spectroscopy

XPS measurements were performed using a Kratos AXIS Ultra X-ray photoelectron spectrometer (Kratos Analytical, Ltd., Manchester, UK). Spectra were acquired using the Al-Kα monochromatic X-ray source (1,486.7 eV). The sample take-off angle was 90°. The vacuum pressure in the analyzing chamber was maintained at 2 × 10^−9^ Torr. High-resolution XPS spectra were collected for F 1 s, C 1 s, and N 1 s peaks with a pass energy of 20 eV and a 0.1 eV step size. Data were analyzed by Kratos Vision data reducing processing software (Kratos Analytical, Ltd.) and Casa XPS (Casa Software Ltd.). Measurements were conducted in triplicates and taken from different regions of each surface. Eventually, the values were averaged.

### Ellipsometry

The peptide coating thickness was measured by an α-SE spectroscopic ellipsometer (J.A. Woollam, Lincoln, Nebraska, USA). Measurements were performed at a wavelength range of 380–900 nm, at a 70° angle of incidence. The optical properties of the substrate were fitted to the stainless steel model. The thickness of the layers and refractive indices were fitted according to the Cauchy model. The coefficients of the Cauchy equation were initially fixed for organic layers (An = 1.45, Bn = 0.01 and Cn = 0). Then, they were allowed to be fitted to determine more accurate values. Measurements were conducted in triplicates; each of the samples were measured three times and the resulting values were averaged.

### Atomic Force Microscopy

The topography of the surfaces was measured by an atomic force microscope (AFM, JPK, Germany) using a SiN_3_ tip (Aspire CT 130-R, Team Nanotech GmbH, Germany) in AC mode. Images were processed by JPK data processing software (JPK Instruments, Germany) and the roughness values were computed from a cross section.

### Quantification of Bacterial Growth on the Surfaces

Initially, a starter bacterial suspension was generated for each of the tested bacteria. Thus, the cells of *B. licheniformis* and *P. aeruginosa* were grown in LB at 37°C in a shaker incubator at 150 rpm for 5 h. Meanwhile, the peptide-coated and uncoated stainless steel surfaces were placed into a sterile 12-well culture plate. Then, 10 μl of the bacterial suspensions were dripped onto the surfaces and incubated for 30 min at room temperature to allow the initial adhesion of bacteria to the surfaces. Afterward, 3 ml of suitable growth medium was added to each plate and the samples were incubated for 18, 42, or 66 h at 37°C. Next, the surfaces were washed using distilled water in order to remove non-adherent bacteria. The surfaces were then transferred into 15-ml test tubes with 1 ml of sterile distilled water and mildly sonicated for 20 s – amplitude, 20%; pulse, 10 s; pause, 10 s – with an ultrasonic processor (Sonics, Newtown, USA). The bacterial counts were detected using the colony-forming unit (CFU) method on LB agar plates that were incubated at 37°C overnight.

### Confocal Laser Scan Microscopy Analysis

Bacterial cells were stained using the FilmTracer LIVE/DEAD Biofilm Viability Kit (Molecular Probes, Eugene, Oregon, USA) according to the manufacturer’s instructions. The surfaces were transferred onto glass slides and visualized with a SP8 confocal laser scanning microscope (CLSM) (Leica, Wetzler, Germany) equipped with a HC PL APO 40x/1. Fluorescence emission from the stained samples was measured at wavelengths of 488 and 552 nm.

### Growth Curve Analysis

Bacteria were first grown in LB with agitation (90 rpm) at 23°C. After 16 h of incubation, the cultures were diluted 1:100 in LB for *P. aeruginosa* and TSB with 0.5% yeast extract for *B. licheniformis.* The diluted samples were then transferred into peptide-coated and uncoated test tubes and incubated for 10 h at 37°C with shaking at 150 rpm. Every 2 h, 1 ml of each sample was collected and the number of viable cells was determined by the CFU method on LB agar plates.

### The Effect of Milk on Peptide Coating Performance Upon Pre-Incubation in Milk

Peptide-coated and uncoated surfaces were placed in 12-well plates. Then, 3 ml of 3% fat homogenized UHT milk was added to each well and the plate was placed in an incubator at 30°C for 16 h. After incubation, the surfaces were washed with distilled water and dried out. Surfaces treated with milk were also examined for their antifouling properties in the same manner as the untreated surfaces.

### The Effect of Peptide Coating on Milk Clotting

Milk-clotting parameters [e.g., the starting time of clotting (min), the curd firmness (V) after 60 min], upon exposure of raw milk to peptide-coated and uncoated test tubes, were measured by Optigraph (Ysebaert, Frepillon, France) (Leitner et al., 2011; Ben-Ishay et al., 2017).

### The Effect of Peptide Coating on Protein Quantity in Raw Milk and Soft Cheeses

Prior to cheese preparation, 50 ml of raw milk samples was stored within either peptide-coated or uncoated test tubes. Rennet enzyme was diluted 1:100 in distilled water, and 2.5 ml of the diluted enzyme was added to each milk sample. The samples were incubated in a water bath at 30°C for 1 h. The resulting cheeses were cut and heated to 40°C for 30 min in a water bath, in order to drain the whey. Then they were transferred into perforated tubes and kept at 4°C overnight to remove the whey. The level of protein in the raw milk and soft cheeses was determined by the Kjeldahl method (Merin et al., 2008).

### Statistical Analysis

Results were subjected to either Student’s *t*-test or Wilcoxon two-sample test, at a significance level of *p* < 0.05, to compare the control and test samples.

## Results

### Coating and Characterizing Stainless Steel Surfaces

Stainless steel is commonly used for fabricating transport pipes and containers in the food processing industry (Gkana et al., 2017). Therefore, we applied the peptide coating on stainless steel surfaces. To allow the peptide to self-assemble on the surfaces, we dipped them in peptide solution (0.5 mg/ml in ethanol) overnight. These conditions were chosen as they resulted in the formation of a peptide layer that would exhibit the best performance against biofilm formation (Maity et al., 2014). The next day, the substrates were thoroughly washed to remove any non-adherent peptide remains and then dried under nitrogen flow.

**Figure 1:**
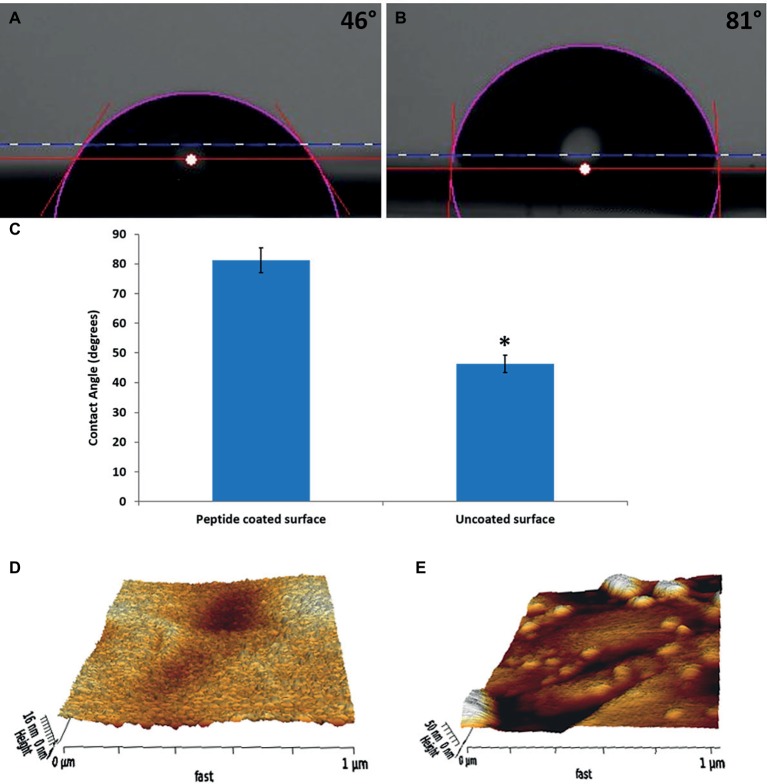
Characterization of the physical properties of the peptide-coated surfaces. Water contact angle images of **(A)** bare stainless steel, **(B)** peptide-coated stainless steel. **(C)** Comparison between the contact angles of the coated and uncoated surfaces. Error bars represent ±SD (*n* = 3). **(D,E)** Surface topography obtained from AFM measurements for **(D)** bare stainless steel and **(E)** peptide-coated stainless steel. ^*^p < 0.05 compared to control (based on Student’s *t*-test).

To confirm that the peptide coating modified the surfaces, we compared the water contact angles of the peptide-coated substrates to those of bare substrates. Coated substrates had an average contact angle of 81 ± 4°, whereas the bare surfaces had a lower angle of 46 ± 3° (Figures 1A–C). The difference between the water contact angle of the bare and peptide-coated surface indicates that the peptide indeed coated the surface and modified it. This increase in the water contact angle value of the peptide-coated surfaces, in line with previous results, also indicates that the modified surfaces exhibited a more hydrophobic nature. These features might contribute to preventing bacterial accumulation on the modified surfaces (Wang et al., 2008; Chapman and Regan, 2012).

XPS confirmed the presence of the peptide on the coated surfaces. Bare surfaces did not display any fluorine signal, whereas the coated surface had 1.5% (atomic concentration) of fluorine on them. In addition, the thickness of the layer was calculated by ellipsometry to be 6 ± 1 nm.

AFM was used to map the topography of the surfaces (Figures 1D,E). The images show a significant difference between the bare and coated substrates. In correlation with previous results (Maity et al., 2014), after coating, the surface was less homogenous with aggregates spread all over it. The roughness of the peptide-coated surface differed from that of the non-coated one on both peaks and valleys and its averaged value (Ra) increased from 1.8 to 2.8 nm.

### Peptide Coating of the Stainless Steel Surfaces Inhibits Biofilm Formation and Maturation

**Figure 2:**
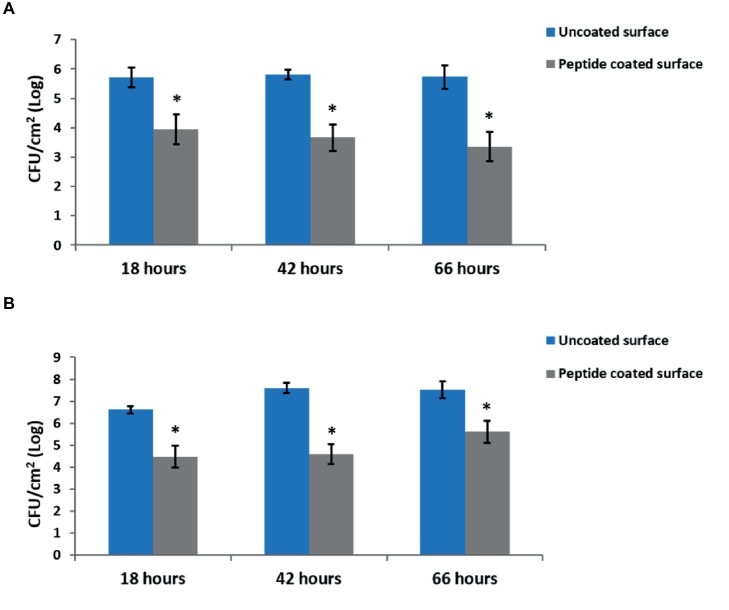
Inhibition of biofilm development by peptide-coated surfaces. Quantification of the number of bacteria adsorbed onto uncoated and peptide-coated stainless steel surfaces at different incubation times for **(A)** *B. licheniformis* and **(B)** *P. aeruginosa*. Error bars represent ±SD (*n* = 9). ^*^p < 0.05 compared to control (based on Wilcoxon two-sample test).

To test the effect of the peptide coating on biofilm formation, we quantified the amount of viable bacteria on peptide-coated and bare stainless steel surfaces by counting the CFUs of the surface-adhered cells. The results show a reduction of around 2-log in the amount of both *B. licheniformis* and *P. aeruginosa* on the peptide-coated surfaces compared with the bare surfaces (Figure 2).

**Figure 3:**
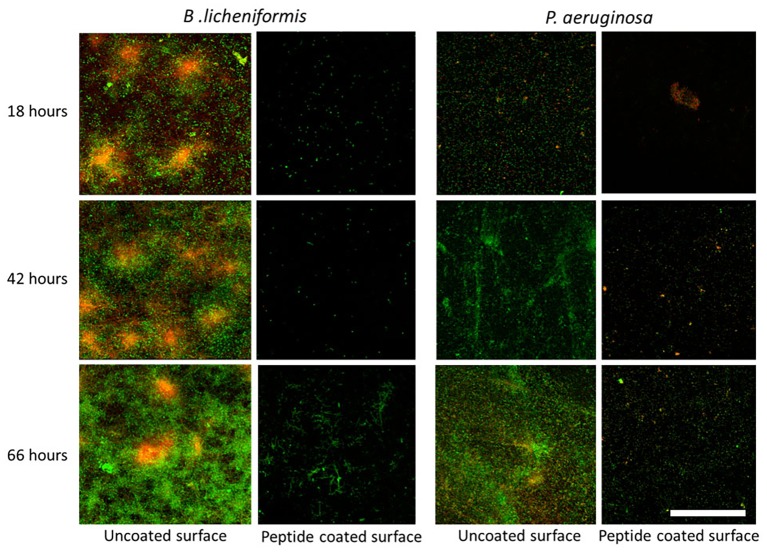
Fluorescent microscopy analysis of inhibition of biofilm establishment using peptide-coated surfaces. CLSM images of biofilms on uncoated and peptide-coated stainless steel surfaces developed at different incubation times. Viable bacterial cells are stained with SYTO 9 green fluorescent nucleic acid stain and dead bacterial cells are stained in red with propidium iodide (PI). On the left panel is biofilm formed by *B. licheniformis* and on the right, *P. aeruginosa* biofilm. The scale bar represents 50 μm.

In addition, we examined the time-course inhibitory ability of the coated surfaces, by measuring the bacterial counts at different time points during biofilm development – from the initial biofilm formation through its maturation. Each time point represents different stages in the biofilm development. The results show a similar percentage in the decrease of biofilm formation following 18, 42, and 66 h of incubation with peptide-coated surfaces in comparison to uncoated surfaces (Figure 2). To further support our results, we analyzed the surface-adhered bacteria using confocal scanning laser microscopy (CSLM) (Figure 3). The images present a significantly lower number of bacterial cells on the peptide-coated surfaces compared with the bare surfaces. This trend was similar for both *B. licheniformis* and *P. aeruginosa*.

### Peptide Coating Is Nontoxic to Bacteria

**Figure 4:**
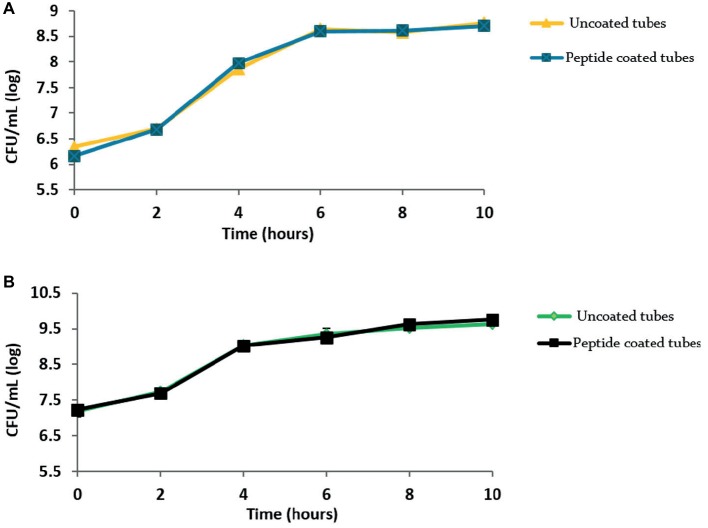
Peptide coating does not affect the growth of bacteria. Growth curves obtained for **(A)** *B. licheniformis* and **(B)** *P. aeruginosa* grown in the presence of peptide-coating and without it. Values were averaged over three repeats.

To rule out any cytotoxic effect of the peptide on the bacteria, we tested the peptide-coated surfaces for their effect on bacterial growth using a growth curve analysis (Figure 4). The curves clearly show similar growth in the peptide-coated vessels in comparison to uncoated ones in both bacterial species.

### Peptide-Coated Surfaces Preserve Their Anti-Biofilm Properties Following Their Exposure to Milk

**Figure 5:**
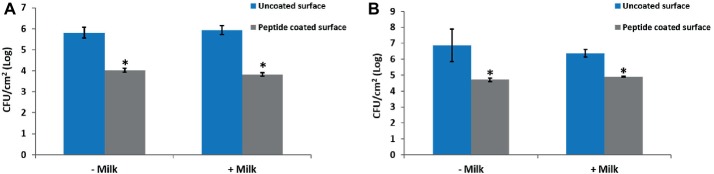
The effect of milk on the anti-biofilm properties of the peptide coating. Peptide-coated stainless steel surfaces exhibit a similar reduction in the number of surface-adhered bacteria with and without pre-incubation in milk. Results for **(A)** *B. licheniformis* and **(B)** *P. aeruginosa.* Error bars represent ±SD (*n* = 4). ^*^p < 0.05 compared to control (based on Wilcoxon two-sample test).

To determine whether exposure of the peptide-coated surfaces to milk impairs the coating stability and anti-biofilm performance, coated and uncoated stainless steel surfaces were incubated in milk overnight prior to their exposure to bacteria. The results show that the biofilm formation was inhibited notably, following bacterial incubation on peptide-coated surfaces compared to uncoated surfaces, regardless of the pre-incubation in milk (Figures 5A,B).

### The Peptide Coating Does Not Affect the Technological Properties of Milk and Its Products

**Figure 6:**
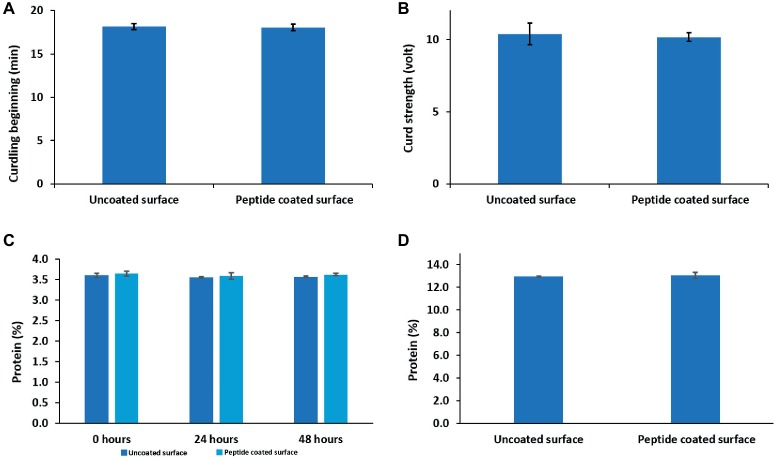
The effects of peptide coating on the technological properties of dairy products. Peptide coating does not induce any change in **(A)** clotting time, **(B)** curd firmness and the amount of protein in **(C)** raw milk and **(D)** soft cheese compared to uncoated surfaces. Error bars represent ±SD (*n* = 9).

One of the requirements of the modified surfaces is to prove that their exposure had no influence on either the quality of milk or its products. Therefore, using an Optigraph instrument, we examined the effect of a peptide-coated surface on milk clotting parameters such as the starting time of clotting (minutes) and the curd firmness (V). The results indicate that the duration of the clotting period of milk exposed to peptide-coated surfaces, 18.0 ± 0.3 min, resembled that of milk from bare samples, 18.1 ± 0.3 min. The strength of the curd obtained under these two conditions was also similar (Figures 6A,B). In addition, we examined how the peptide coating affected the protein levels in raw milk as well as the effect of incorporating proteins into cheese using the Kjeldahl method. Importantly, we could not detect any changes in the amount of protein in milk as well as in the cheese samples (Figures 6C,D).

## Discussion

Bacterial adhesion to surfaces and the formation of biofilms in dairy processing equipment are the main source of contamination of dairy products (Cappitelli et al., 2014). The use of biocides to eradicate bacterial biofilms is not advised because they could be released to the products (Midelet and Carpentier, 2004). Therefore, it is desirable to prevent the adhesion of bacteria in advance in order to mitigate subsequent biofilm formation. This study provides evidence of the possibility of successfully modifying milk contact surfaces such as stainless steel to prevent biofilm formation and, thus, to prevent subsequent contamination of milk during its processing.

Unlike early stage biofilms, mature biofilms consist of a developed extracellular matrix and more protected bacterial cells. Therefore, it is important to develop anti-biofilm coatings that can reduce bacterial levels for long durations. Similar to the short incubation time (18 h), a lower number of bacteria were counted and sparse bacteria could be detected for the peptide-coated surfaces at 42- and 66-h time points. As expected, with increasing incubation times, bare stainless steel surfaces exhibited denser biofilms with growing thickness, whereas the density of bacterial cells on peptide-coated surfaces was significantly low and remained constant throughout time (Figures 2 and 3). These results indicate that the peptide-coated surfaces could be effective against biofilm formation and maturation for both Gram-positive and Gram-negative bacterial species.

Although biofilm prosperity is compromised in the presence of the peptide coating, apparently the bacteria are not directly affected by it. Growth curves showed that the tested bacterial species were able to thrive despite the presence of the peptide coating. Thus, we concluded that the peptide-coated surfaces were not cytotoxic to the tested bacterial strains. This conclusion is also supported by CLSM micrographs (Figure 3) showing that very few bacteria adhered to the peptide-coated surfaces; however, most of them were found to be alive (since they were stained in green). These results support our conclusion of an anti-biofilm mode of action of the peptide coating rather than of its biocidal activity.

The results presented in Figures 5A,B suggest that the peptide coating properties remained intact after incubation with milk, keeping not only the same trend but also the same order of magnitudes in resisting bacterial accumulation. In the dairy industry, coating effectiveness and stability may decrease owing to the presence and/or adsorption of milk proteins (Oulahal-Lagsir et al., 2003; Yuan et al., 2018). These proteins may adhere to the surface and act as a conditioning film on which bacterial adherence could be promoted (Sjollema et al., 2018). Considering that the coating is not affected by the presence of milk, together with the biofilm’s inhibition with time, and the results obtained for the growth curves, implies that the peptide coating is stable under these different conditions.

The process by which milk coagulates into curd might be sensitive to the environment. Different components in the surroundings may affect the timing of clotting, and curd firmness (Munro et al., 1984). Since it is possible that the milk components would react with the peptide coating, this could have a drastic impact on the quality of the curd and its nutritious properties (Leitner et al., 2011). None of the parameters tested displayed any difference compared to the results obtained from the non-coated samples, implying that the peptide coating did not affect the technological properties of the dairy products. More importantly, there was no change in the protein concentration in milk or cheese following the exposure of the raw milk to the coated surface.

## Conclusions

In this paper, we demonstrated that a modification of the stainless steel surface using a peptide-based coating prevents biofilm formation and subsequent maturation by bacterial species prevalent in the dairy industry. In addition, we found that the peptide coating does not affect the technological properties of dairy products; thus, it can be an attractive solution and can be safely used in the dairy industry or in the manufacture of various associated dairy products.

## Author Contributions

MS and MR designed and planned the study. SN synthesized the antifouling peptide, coated the stainless steel surfaces, and performed the physical characterization of the peptide-coated surface. AF performed the experiments related to the characterization of the anti-biofilm properties of the surfaces as well as the technological effect of the surfaces following their exposure to raw milk. MS and AF wrote the initial manuscript. AF and SN contributed equally to this work. All authors integrated the data, discussed the results, and crafted the final manuscript.

### Conflict of Interest Statement

The authors declare that the research was conducted in the absence of any commercial or financial relationships that could be construed as a potential conflict of interest.
